# Intercolony variation in reproductive skipping in the African penguin

**DOI:** 10.1002/ece3.9255

**Published:** 2022-09-06

**Authors:** Freddie W. Leith, Jennifer L. Grigg, Barbara J. Barham, Peter J. Barham, Katrin Ludynia, Cuan McGeorge, Andile Mdluli, Nola J. Parsons, Lauren J. Waller, Richard B. Sherley

**Affiliations:** ^1^ Centre for Ecology and Conservation College of Life and Environmental Sciences, University of Exeter Penryn UK; ^2^ Penguin Datasystems Bristol UK; ^3^ H. H. Wills Physics Laboratory University of Bristol Bristol UK; ^4^ Department of Biodiversity and Conservation Biology University of the Western Cape Cape Town South Africa; ^5^ Southern African Foundation for the Conservation of Coastal Birds (SANCCOB) Cape Town South Africa; ^6^ Department of Biological Sciences University of Cape Town Cape Town South Africa; ^7^ Stony Point Nature Reserve, CapeNature Betty's Bay South Africa; ^8^ Robben Island Museum, Robben Island South Africa; ^9^ FitzPatrick Institute of African Ornithology, DST‐NRF Centre of Excellence University of Cape Town Cape Town South Africa

**Keywords:** Benguela ecosystem, breeding propensity, mark‐recapture, population dynamics, seabirds, survival

## Abstract

In long‐lived species, reproductive skipping is a common strategy whereby sexually mature animals skip a breeding season, potentially reducing population growth. This may be an adaptive decision to protect survival, or a non‐adaptive decision driven by individual‐specific constraints. Understanding the presence and drivers of reproductive skipping behavior can be important for effective population management, yet in many species such as the endangered African penguin (*Spheniscus demersus*), these factors remain unknown. This study uses multistate mark‐recapture methods to estimate African penguin survival and breeding probabilities at two colonies between 2013 and 2020. Overall, survival (mean ± *SE*) was higher at Stony Point (0.82 ± 0.01) than at Robben Island (0.77 ± 0.02). Inter‐colony differences were linked to food availability; under decreasing sardine (*Sardinops sagax*) abundance, survival decreased at Robben Island and increased at Stony Point. Additionally, reproductive skipping was evident across both colonies; at Robben Island the probability of a breeder becoming a nonbreeder was ~0.22, versus ~0.1 at Stony Point. Penguins skipping reproduction had a lower probability of future breeding than breeding individuals; this lack of adaptive benefit suggests reproductive skipping is driven by individual‐specific constraints. Lower survival and breeding propensity at Robben Island places this colony in greater need of conservation action. However, further research on the drivers of inter‐colony differences is needed.

## Graphical abstract

This study utilises mark‐recapture methods to evaluate population dynamics in the endangered African penguin across two colonies. Alongside finding key inter‐colony differences in survival and breeding probabilities, we provide here the first evidence of reproductive skipping behaviour in the African penguin.

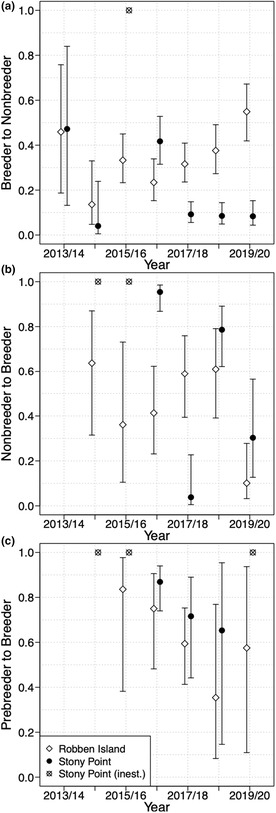

## 1 INTRODUCTION

Survival and reproduction are two key demographic processes for all organisms, yet both are energetically costly and use the same limited resources (Stearns, 1992; Williams, 1966). High investment in reproduction 1 year diverts energy away from other processes, potentially reducing future survival probabilities and, particularly in long‐lived species, impacting on an individual's lifetime reproductive output (Williams, 1966). Long‐lived species therefore often act as ‘prudent parents’, prioritizing survival over reproduction (Cam et al., 1998; Sæther et al., 1993). This strategy is widespread among long‐lived vertebrates (e.g., Bleu et al., 2016; Rivalan et al., 2005; Romine et al., 2009), especially pelagic foraging seabirds (Jouventin & Dobson, 2002; Le Bohec et al., 2007; Sanz‐Aguilar et al., 2011), which regularly undertake long and energetically costly foraging trips. However, these nonbreeders are often not accounted for in population studies, despite their ability to obscure low population growth rates and reduce the reliability of population estimates (Lee et al., 2017).

The drivers of reproductive skipping are predominantly grouped into adaptive and non‐adaptive explanations. Adaptive explanations suggest animals actively avoid reproduction when conditions are poor (e.g., low food availability; Gauthier‐Clerc et al., 2001). This process enhances survival probability, ensuring future breeding opportunities and improving lifetime reproductive output. Alternatively, reproductive skipping may be driven by non‐adaptive individual constraints, e.g., unavoidable events like pair‐bond breakdown or forced nest site relocation (Bradley et al., 2000; Jeschke et al., 2007; Salas et al., 2020). Additionally, the intrinsic quality of animals will vary regardless of these mechanisms, with some ‘higher quality’ individuals consistently achieving higher survival and reproductive rates (Cam et al., 1998; Jenouvrier et al., 2015). In reality these mechanisms co‐occur. For example, ‘lower quality’ individuals are more likely than ‘high quality’ individuals to adaptively skip reproduction under poor conditions (Robert et al., 2012; Souchay et al., 2018). The extent and drivers of reproductive skipping therefore vary not only between species and populations, but also at the individual level. Characterizing inter‐population variation in reproductive skipping is therefore vital to better understand localized population dynamics and to identify areas in need of conservation action. However, in many species reproductive skipping is yet to be evaluated; the endangered African penguin (*Spheniscus demersus*) is a key example of this, and the focus of our study.

The African penguin is endemic to the Benguela upwelling system, where they currently breed at 28 localities in Namibia, South Africa's Western Cape, and South Africa's Eastern Cape (Sherley et al., 2020). Breeding is based around social monogamy and strong breeding site fidelity, with >90% of birds historically expected to retain the same mate in the absence of partner mortality (Crawford et al., 1995). Since 1989, the African penguin population has declined by almost 65% (Sherley et al., 2020). While historically driven by egg collection and guano scraping (Crawford et al., 2018; Frost et al., 1976), current declines are predominantly attributed to reduced prey availability (Crawford et al., 2011; Sherley et al., 2020). Shifting geographic distributions of sardine (*Sardinops sagax*) and anchovy (*Engraulis encrasicolus*), the main prey of African penguins, apparently exacerbated by competition with fisheries, has been linked to both reduced survival (Robinson et al., 2015; Sherley, Abadi, et al., 2014) and lower breeding success (Crawford, Barham, et al., 2006; Sherley et al., 2013, 2021). However, population growth of seabirds can also be influenced by the proportion of the population that breeds each year (Cam et al., 1998; Le Bohec et al., 2007). Previous demographic models for the African penguin have made assumptions about the proportions of mature females available to breed each year, ranging from 0.83 to 1.00 (Shannon & Crawford, 1999; Sherley et al., 2018; Weller et al., 2016). As African penguin colonies continue to decline to levels at which Allee effects may manifest themselves (Ryan et al., 2012), characterizing the presence and drivers of reproductive skipping is vital to improve our understanding of local population dynamics and to guide future conservation decisions.

Here we use mark‐recapture data from two African penguin colonies in the Western Cape, spanning an 8‐year period (2013–2020). Changes in apparent survival and breeding propensity were examined over time and between colonies, with a focus on understanding the presence and trends in reproductive skipping behavior. The effects of food availability on survival and breeding propensity were also examined, in line with previous work linking food availability to changes in survival and breeding success in this species (e.g., Crawford et al., 2011).

## 2 MATERIALS AND METHODS

### 2.1 Study site and data collection

**FIGURE 1:**
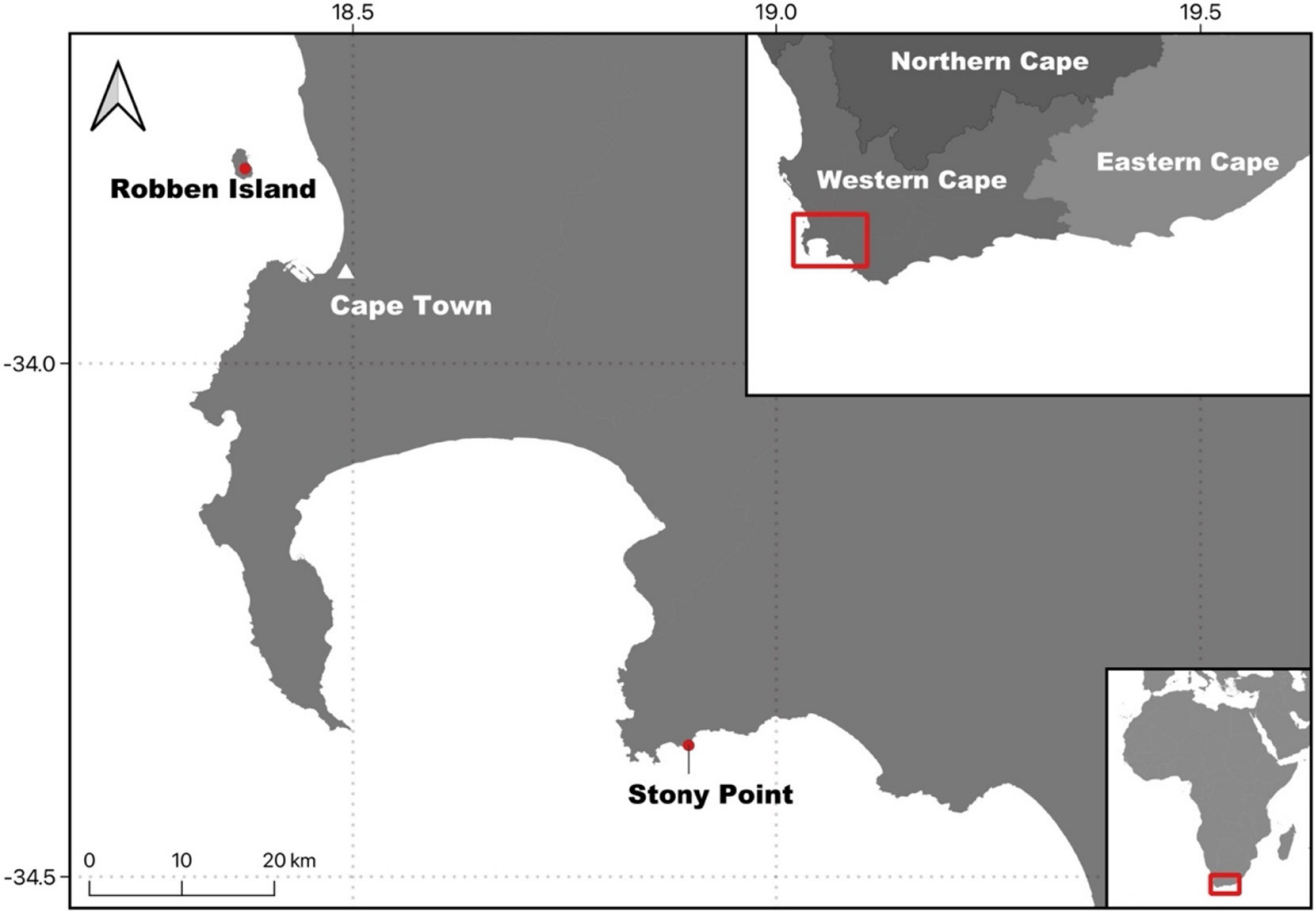
Study site locations (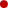) at Robben Island and Stony Point in the Western Cape, South Africa, in relation to Cape Town (△).

Data collection took place between 2013 and 2020 at two African penguin colonies in the Western Cape Province, South Africa: Robben Island (33°48′S, 18°22′E) and Stony Point (34°22′S, 18°53′E; Figure 1). From 2013 onwards, penguins were captured in each colony, and injected with passive integrated transponders (PITs). For 2013 and 2014 these were Half Duplex (HDX), 134.2 kHz, ISO 11784/11785 compliant, 32 mm glass PITs (31.2 [l] × 3.85 [d] mm, weight 0.8 g), injected subcutaneously into the back of the neck. From 2015, Full Duplex (FDX‐B), 134.2 KHz, ISO compliant, 12 mm PITs (Biomark, Boise, ID, USA) were injected subcutaneously into the skin flap posterior to the left leg. Subsequent encounter data of tagged penguins were then collected from 2014 onwards. As part of routine nest monitoring between March and October, the presence and breeding status (breeding or nonbreeding) of tagged birds was identified using a hand‐held transponder reader (Datamars model GES3SEU with external stick antenna from 2014–2015, Allflex model RS420 from 2016 onwards). Each year captured untagged birds were tagged under the same protocol (Table S1: Appendix S2). Supplementing this, ground reader systems (Biomark IS1001 with loop antenna) were installed across commonly used highways to/from the sea; one reader was installed at Robben Island in 2015, and two at Stony Point in 2016 and 2017 respectively. Although not completely curtailed in either season, data collection using the hand‐held transponder readers was negatively impacted by an Avian Influenza outbreak in 2018 (Molini et al., 2019), which limited close approaches by researchers to penguin nests, and by the COVID‐19 pandemic in 2020, which limited the number of person‐days spent in the field (relative to other breeding seasons), particularly at Robben Island.

### 2.2 Encounter data

We used mark‐recapture data from 899 adult penguins, 387 at Robben Island and 512 at Stony Point (Table S1). Encounter data were collected over the breeding season (March–October); apparent survival rates consequently refer to the nonbreeding season (November–February). We assigned each penguin a state both during initial marking and each subsequent encounter. Individuals were considered breeders if they were attending chicks or eggs in a nest site. These nest inspections only cover a relatively small area of the whole breeding colony at both sites (see e.g. Sherley, Barham, et al., 2014), but reflect the areas in which most monitoring activity (including the initial marking of birds with PITs) occurs. Additionally, because detection of breeders on nest inspections will not be perfect, if individuals were encountered in the colony via the ground reader ≥6 times over a minimum of 12 days and a maximum of 120 days but not confirmed as breeders during nest inspections, they were considered breeders based on knowledge of African penguin breeding biology (Williams & Cooper, 1984). The number of these individuals recorded as breeding purely based on the ground reader, relative to those confirmed breeding during nest inspections was recorded annually in each colony (Table S2: Appendix S2).

Individuals were defined as nonbreeders either if they were observed in the colony as nonbreeders (never encountered with eggs/chicks or encountered <6 times as per above), or if they were not encountered in a given year, but reobserved in a following year. Despite the inclusion of the ground reader data, we cannot completely rule out the possibility that nonbreeders might have included some birds that nested but lost the clutch quickly, either before the reproductive attempt was detected on nest inspections or before they were recorded ≥6 times on the ground reader. To distinguish between birds encountered as nonbreeders that were apparently skipping reproduction and young birds yet to breed for the first time, we included an additional state of ‘prebreeder’, assigned to any birds marked/encountered as nonbreeders that had not yet been encountered breeding within their encounter histories.

Earlier tagging of African penguins used stainless steel flipper bands (Sherley, Abadi, et al., 2014). However, these have now been phased out due to potential data bias, both through human error, such as incorrectly reading tags, and potential deleterious effects on penguin survival and behavior (Dann et al., 2014; Dugger et al., 2006). Within this dataset, four double‐marked (flipper band from previous tagging, and transponder from this study) individuals were removed to reduce any potential bias and one individual encountered breeding at >1 colony was removed from the dataset as inter‐colony movement was not the focus of our study. While movement between colonies is extremely rare in breeding African penguins, younger birds do disperse (Sherley, Abadi, et al., 2014) and PITs can fail (e.g., Dann et al., 2014). Permanent emigration outside of the study area and failure or loss of the PIT tags would be indistinguishable from mortality, so here we estimate apparent rather than true survival.

### 2.3 Fish biomass data

African penguins breed in the austral winter, during which time they feed within <50 km of their breeding colonies (Campbell et al., 2019; Pichegru et al., 2013). But during the nonbreeding season in the austral summer, they undergo a catastrophic molt (Crawford, Hemming, et al., 2006), before and after which they forage over a much wider area (up to 600 km away from their colonies, Carpenter‐Kling et al., 2022). The acquisition of sufficient body reserves during this nonbreeding period is likely to be key for survival and for decisions about breeding in the next season (Sherley et al., 2013; Wolfaardt et al., 2009). Thus, to determine the impact of food availability on survival and breeding probabilities, we used estimates of sardine and anchovy spawner (fish aged ≥1 year) biomass from hydro‐acoustic surveys conducted by the Department of Forestry, Fisheries, and the Environment (DFFE) in November each year between 2013 and 2019 (Coetzee et al., 2020; Coetzee & Merkle, 2019). For detailed survey methods see Coetzee et al. (2008). We related biomass to survival using the biomass from the survey at the start of the nonbreeding period (i.e., survival from the 2013 breeding season to the 2014 breeding season was related to the biomass estimate for November 2013), and considered the biomass of the two species (sardine and anchovy) combined and for each separately in our modeling framework. And, finally, although the portion of the biomass estimated to occur west of Cape Agulhas (Hondeklip Bay to Cape Agulhas), has previously been linked to measures of penguin survival and reproduction at Robben Island (e.g., Robinson et al., 2015; Sherley et al., 2013), here we chose to use the estimates from the full survey area (Hondeklip Bay to Port Alfred, see Coetzee et al., 2020) as Stony Point is closer to Cape Agulhas than Robben Island and recent tracking data shows that nonbreeding penguins do forage to the east of Cape Agulhas (Carpenter‐Kling et al., 2022).

### 2.4 Multistate mark‐recapture models

To estimate the probabilities of survival (*Φ*), encounter (or recapture, *ρ*), and transition (*ψ*) between states (breeder, non‐breeder, and prebreeder), multistate mark‐recapture models were constructed using program MARK and the ‘RMark’ package in R (Laake, 2013; White & Burnham, 1999). Within these models, a group effect for colony (Robben Island and Stony Point) was included to evaluate colony differences in the estimates. Known parameters were fixed to improve model performance; since only breeders were marked in 2013 across both colonies (with nonbreeders marked in subsequent years, Table S1), survival and transition rates for nonbreeders and prebreeders were fixed to zero during 2013–14, as was recapture in 2014 in both colonies. Additionally, no nonbreeders were marked in 2014 at Robben Island, so prebreeder survival and transition during 2014–15, and prebreeder recapture in 2015 were also fixed to zero for this colony. Finally, impossible transitions *ψ*(prebreeder to nonbreeder), *ψ*(nonbreeder to prebreeder), and *ψ*(breeder to prebreeder) were fixed to zero at both colonies.

Initially, a general model was developed assuming time, state, and colony dependence for survival, recapture, and transition probabilities (model A8, Table S4). Simpler model structures were also tested for recapture whereby years were pooled into two groups to represent before and after ground readers were installed in each colony. For survival and transition, simpler models were also included whereby time dependence was replaced with both combined and separated annual sardine and anchovy spawner biomass to determine whether fish abundance offered better predictive power for survival and transition probabilities than the fully time‐dependent model.

Recapture probabilities were modeled first, with the best fitting model taken forward to assess survival probabilities, followed by transition. Model selection was based on Akaike's Information Criterion corrected for over‐dispersion and small sample size (QAICc; Lebreton et al., 1992). When models differed by QAICc < 2, they were considered approximately equivalent (Burnham & Anderson, 2002), and the model with the lowest number of parameters was considered the most parsimonious. Goodness‐of‐fit (GOF) tests for the general model (JMV model; Pradel et al., 2003) were performed using package ‘R2ucare’ (Gimenez et al., 2018) in Program R, with $\hat{c}=\chi^{2}/\mathrm{df}$, where df = the degrees of freedom.

## 3 RESULTS

The overall GOF test for the general (JMV) model showed significant lack of fit to the data ($\chi_{65}^{2}$ = 115.62, *p* < .001, detailed results in Table S3 in the Appendix S2). This lack of fit was accounted for during model selection using a variance inflation factor ($\hat{c}$ = 1.78).

### 3.1 Encounter

**FIGURE 2:**
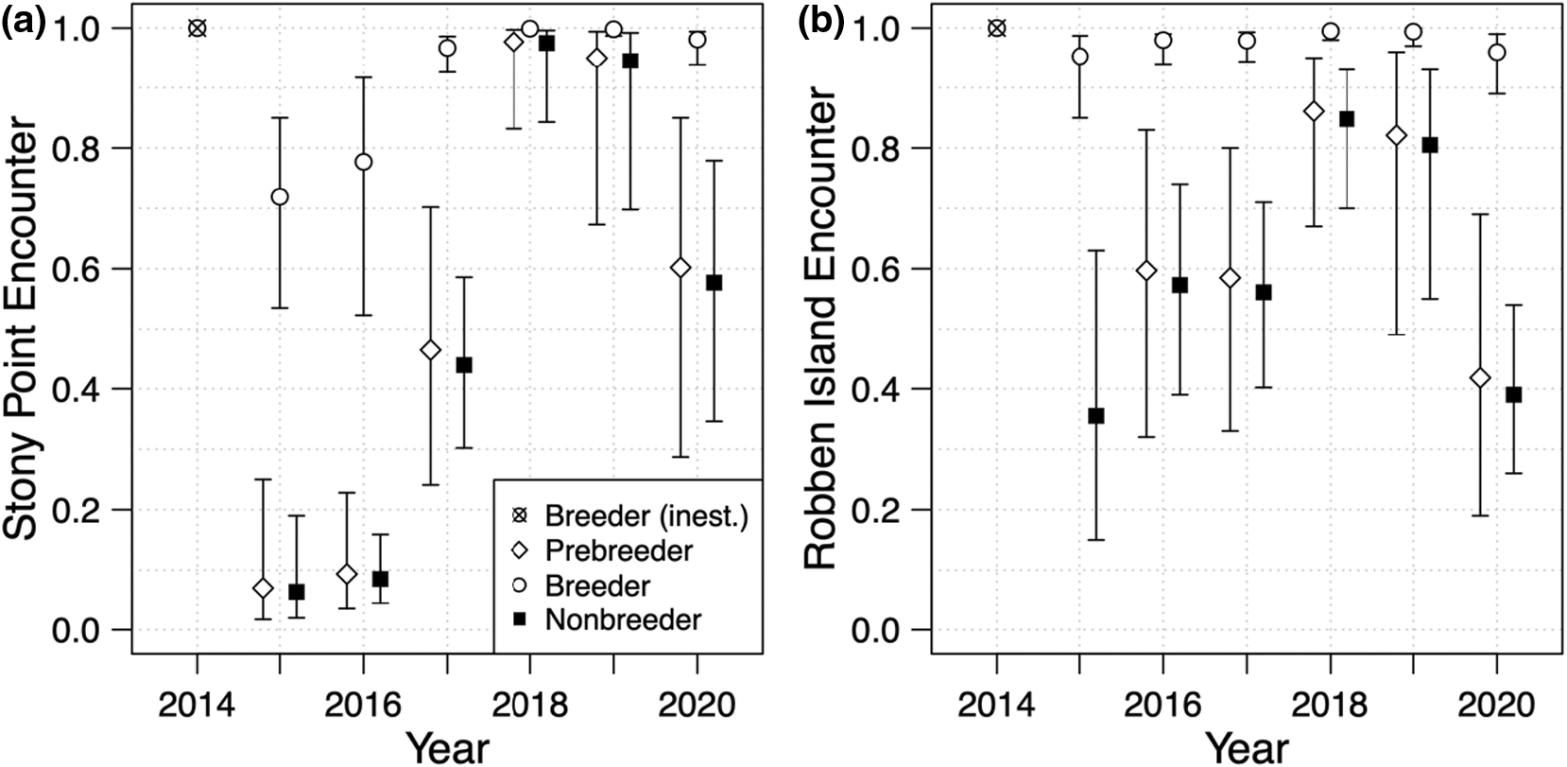
Time‐dependent encounter probabilities for adult African penguins at (left) Stony Point and (right) Robben Island between 2014 and 2020, taken from the best overall model (model C36, Table S4). Error bars represent 95% confidence intervals.

The best‐supported model for encounter (model A10, Table S4 in the Appendix S2) included an interactive effect of time and colony (Robben Island and Stony Point), and an additive effect of state (breeder, nonbreeder, and prebreeder). At Stony point, encounter rates appeared lower during 2015 and 2016, increasing up to 2018 and 2019, followed by a decrease in nonbreeder and prebreeder encounter in 2020 (Figure 2). Meanwhile at Robben Island, a general increase in encounter probability for nonbreeders and prebreeders was evident between 2015 and 2018, followed by a decrease, while breeder encounter rates remained consistently high (Figure 2). Regardless of colony, the probability of encountering breeders was consistently higher than nonbreeders and prebreeders, with all estimable breeder encounter estimates >0.9 at Robben, and 4 out of 6 estimates >0.9 at Stony Point.

### 3.2 Survival

**FIGURE 3:**
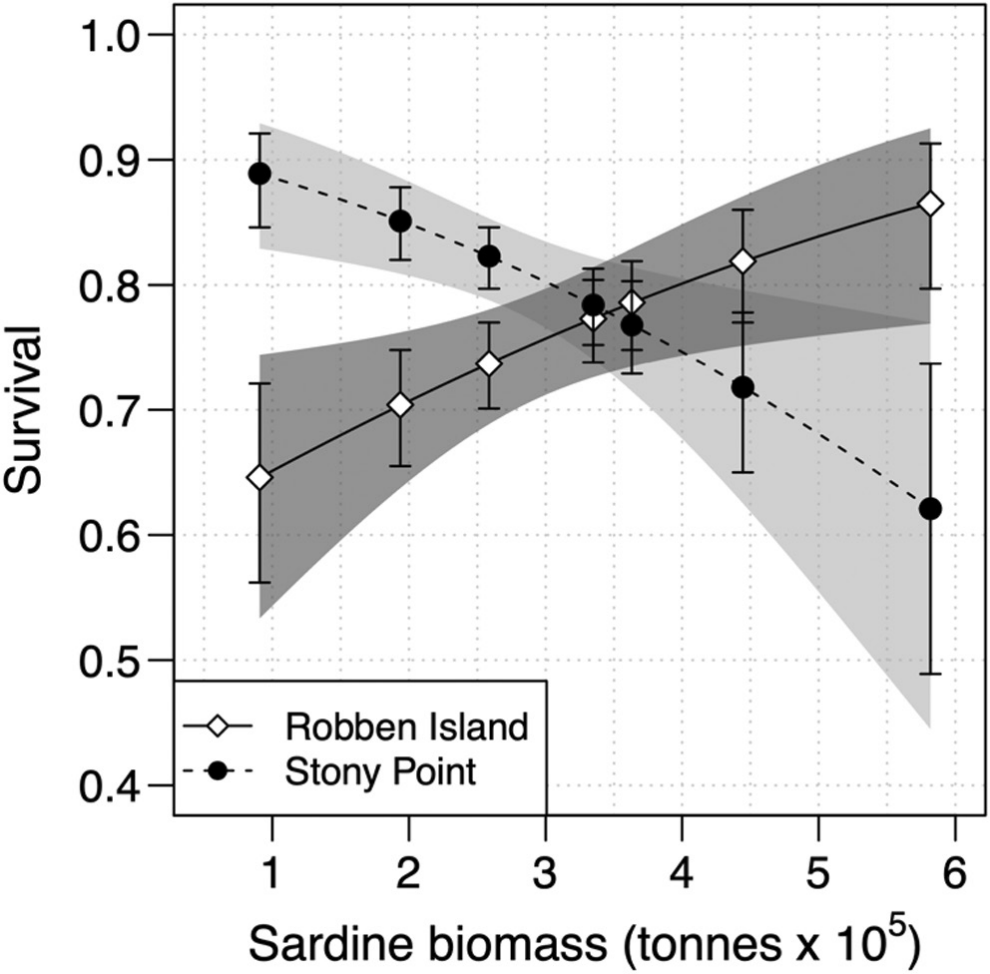
Observed (points) and predicted (black lines) survival probabilities of adult African penguins at Robben Island and Stony Point in relation to annual Sardine spawner biomass, taken from the best overall model (model C36, Table S4). Error bars and dotted lines represent 95% confidence intervals of observed and predicted estimates respectively.

Annual apparent survival was variable at both colonies, ranging from 0.62 to 0.89 at Stony Point, and 0.65–0.87 at Robben Island. Overall survival (±*SE*) was slightly higher at Stony Point (0.82 ± 0.01) than at Robben Island (0.77 ± 0.02) based on a constant model. However, the best‐supported survival model (model B22, Table S4 in the Appendix S2) contained an interaction between sardine spawner biomass and colony; the relationship was positive at Robben Island, but negative at Stony Point (Figure 3). There was some support for an interaction between time and colony (model B32, Table S4 in the Appendix S2), but this model produced a higher QAICc and contained more parameters, and so was not retained as the best model. Some support was shown for an additive effect of state on survival (model B24, Table S4 in the Appendix S2), with breeding individuals exhibiting slightly higher survival rates, but this was not retained in the best model.

### 3.3 Transition

**FIGURE 4:**
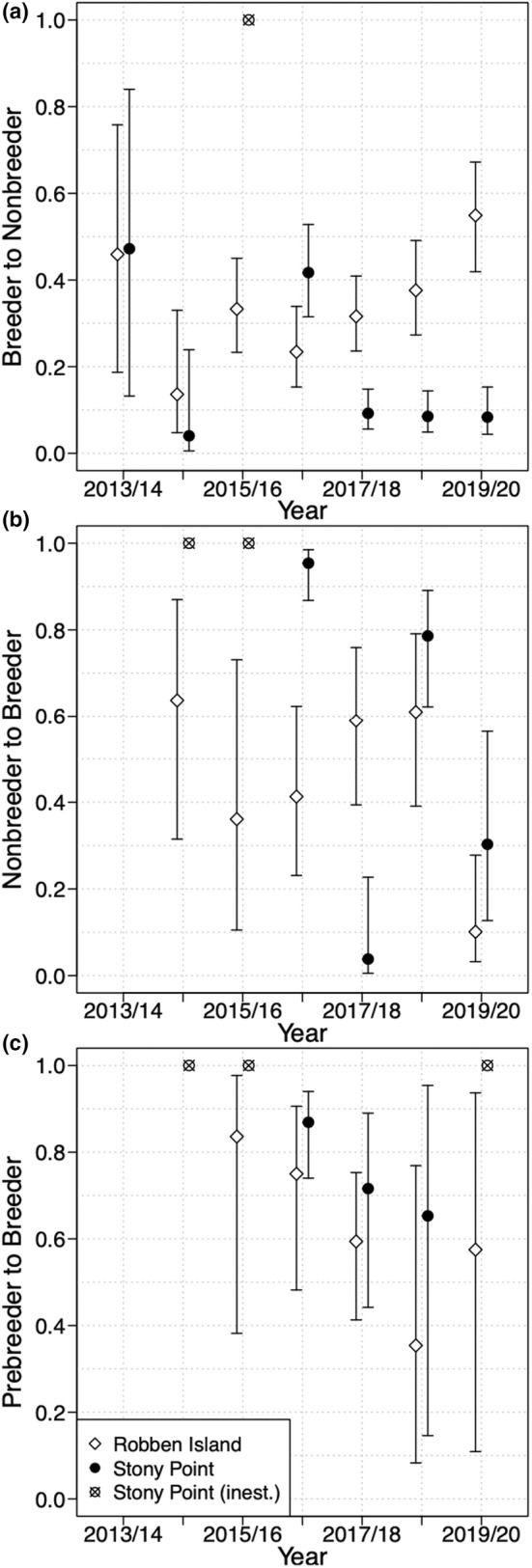
Time‐dependent probability of transition out of (top) breeder, (middle) nonbreeder, and (bottom) prebreeder states for adult African penguins at Stony Point and Robben Island between 2013–14 and 2019–20, taken from the best overall model (model C36, Table S4). Error bars represent 95% confidence intervals.

Strong support for a three‐way interaction between time, colony and state on transition probabilities was evident from the model selection (model C36, Table S4 in the Appendix S2). At both colonies, breeding individuals were more likely to breed again than to skip reproduction the following year except in 2019/20 at Robben Island (Figure 4a). However, breeding individuals were generally more likely to skip reproduction at Robben Island than at Stony Point. Individuals at Robben Island also showed an increasing prevalence for reproductive skipping from 2014 onwards (Figure 4a). Colony differences were especially evident between 2017/18 and 2019/20, with all estimates at Robben Island > 0.3, whereas estimates at Stony Point were all <0.1 (Figure 4a).

Transitions out of the nonbreeding state were more variable and less accurately estimated (Figure 4b). At Robben Island, the probability of nonbreeding birds becoming breeders increased between 2015/16 and 2018/19, followed by a decrease in 2019/20, but estimates at Stony Point showed no clear trend. Overall, nonbreeders were more likely to breed the following year than skip reproduction again (overall probability ±*SE* = 0.66 ± 0.06 at Robben Island and 0.69 ± 0.06 at Stony Point, based on a constant model). However, individuals skipping reproduction were still generally less likely to breed the next year than breeding individuals (Figure 4).

Finally, transition estimates for prebreeders were also poorly estimated, because of the small number of individuals in this state, especially during the earlier years of the study. However, the estimates suggest a general decrease in the probability of transitioning into a breeding state over time at both colonies, but with prebreeders consistently more likely to breed the following year at Stony Point than at Robben Island (Figure 4c).

## 4 DISCUSSION

Reproduction and survival are key demographic parameters impacting population dynamics (Stearns, 1992). We used mark‐recapture techniques to evaluate these parameters for the African penguin at two colonies and confirmed the presence of reproductive skipping behavior. We illustrate that variation in survival is linked to food availability, with lower sardine abundance linked to lower survival at Robben Island, and higher survival at Stony Point. Similarly, inter‐colony differences were evident in breeding propensity; reproductive skipping occurred at both colonies, but at a higher rate at Robben Island than at Stony Point. Alongside providing key demographic information on the endangered African penguin, this study represents the first reliable estimates of reproductive skipping in this species.

### 4.1 Survival

Adult survival of seabirds is generally high (Dias et al., 2019). Long‐term mean adult survival rates for penguins usually exceed 0.8, with most (9 of 13 species reported) above 0.85 (Bird et al., 2020). Even in a population of Magellanic penguins (*S. magellanicus*), declining at ~1.3% per annum, adult survival was >0.8 in 23 of 25 study years (92%; Gownaris & Boersma, 2019). Based on this, African penguin survival rates in this study appear to be relatively low compared to other penguin species and are on average below the level needed to keep the population in equilibrium (~0.85–0.88, Crawford, Barham, et al., 2006), especially at Robben Island (0.77 ± 0.02) where survival was only >0.8 in 2 of 7 years. However, our estimates remain consistent with previous annual survival estimates for adult African penguins which have ranged between 0.47 and 0.99 in the past and averaged about 0.74 since 2004 (Sherley, Abadi, et al., 2014; Sherley et al., 2018; Wolfaardt et al., 2008). They also represent the first survival estimates from African penguins where no individuals were tagged with potentially harmful flipper bands. Within this study, we found that changes in survival are predominantly driven by changing prey (sardine) abundance, with lower abundance underpinning lower survival at Robben Island and higher survival at Stony Point. Understanding the drivers of these colony‐specific differences is fundamental to the successful future population management of African penguins.

The finding that survival declines with reductions in with sardine abundance at Robben Island is consistent with previous long‐term relationships in the literature (Robinson et al., 2015; Sherley, Abadi, et al., 2014). This decline in survival underlines the recent concern for the long‐term viability of the colony at Robben Island (Sherley et al., 2018), given the low and declining availability of sardine to seabirds off western South Africa (Crawford et al., 2019; Robinson et al., 2015). However, the increasing survival rates with decreasing food abundance in individuals at Stony Point are more surprising. This apparent negative relationship may be explained by the presence of additional factors, such as predation and/or density dependence (Appendix S1), which can impact survival differently across colonies (Weller et al., 2016). A similar idea has been suggested for nearby Dyer Island, where pressures other than food availability (mainly predation) appeared to become more important as the population fell below 3500 pairs and may now may hold the population at a low level (Ludynia et al., 2014; Weller et al., 2016). In other words, food availability may not currently be the dominant external driver of variation in survival at Stony Point.

Alternatively, this may be explained by limitations within our analysis; for example, the fisheries data we used index fish abundance across most of South Africa's coastal waters (Coetzee et al., 2008) and may not necessarily adequately reflect localized food availability at both colonies (Sherley et al., 2013). Eastward displacement of sardine and anchovy in the Benguela upwelling system is driving decreased food availability for seabirds in the Western Cape (Crawford, 2007; Crawford et al., 2019) and making it more difficult for birds breeding to the north of Cape Town to access prey resources before and after molt than birds breeding at Stony Point (Carpenter‐Kling et al., 2022). Seabirds often show non‐linear or threshold responses to prey abundance (e.g., Cury et al., 2011; Sherley et al., 2013), and localized food availability may stay consistently higher throughout the year around Stony Point than around Robben Island because of this displacement, breaking the link between very low prey biomass and survival. Or, prey abundance/availability may interact with predation (Strydom et al., 2022) or density dependence (Sherley, Barham, et al., 2014; Appendix S1) in ways we do not yet fully understand. Further research with colony‐specific estimates of prey availability (Campbell et al., 2019) and longer time‐series of survival rates will be needed to tease apart the various potential drivers of inter‐colony differences in survival.

### 4.2 Reproductive skipping: adaptive or nonadaptive?

Our results also indicate that African penguins are not breeding as often as theoretically possible, implying they are either making adaptive decisions to avoid reproductive costs some years, and/or that individual‐specific constraints are limiting the ability to breed each year in some individuals. Under adaptive explanations, reproductive skipping should be beneficial, increasing survival and/or future breeding probabilities (Williams, 1966). On the contrary, our results show that individuals skipping reproduction had a lower probability of breeding the next year and no survival gain compared to breeding individuals. This is consistent with reproductive skipping in African penguins being driven by non‐adaptive individual‐specific constraints, with higher quality individuals being more likely to breed and remain breeders the following year (Jenouvrier et al., 2015; Lescroël et al., 2009).

Supporting this, inter‐individual differences in physiology and behavior of African penguins are known. For example, some individuals travel further and dive more often (Campbell et al., 2019; Traisnel & Pichegru, 2019), which may indicate inter‐individual differences in foraging efficiency. More efficient foragers may be better able to meet the energetic requirements of reproduction (sensu Lescroël et al., 2010). Other individual‐level traits can also influence breeding success, like aggression (Traisnel & Pichegru, 2018) or age (Kappes et al., 2021), and may also interact with individual quality to affect reproductive skipping. More experienced or higher quality individuals may also be better placed to retain mates or nest sites, or to find a new breeding partner after divorce or mate mortality (Bruinzeel, 2007). The lower survival rates at Robben Island coincided with higher probabilities of reproductive skipping, which may imply a higher incidence of mate loss as the global penguin population declines. And there is some evidence for a male‐biased sex ratio in the African penguin population (Spelt & Pichegru, 2016), which may contribute to reproductive skipping if males are consequently less likely to find a mate following divorce or mate mortality. Ultimately, however, while our study provides good evidence for reproductive skipping in African penguins, disentangling the possible proximate and ultimate mechanisms will require further individual‐level monitoring in future.

### 4.3 Inter‐colony variation in breeding probability

**FIGURE 5:**
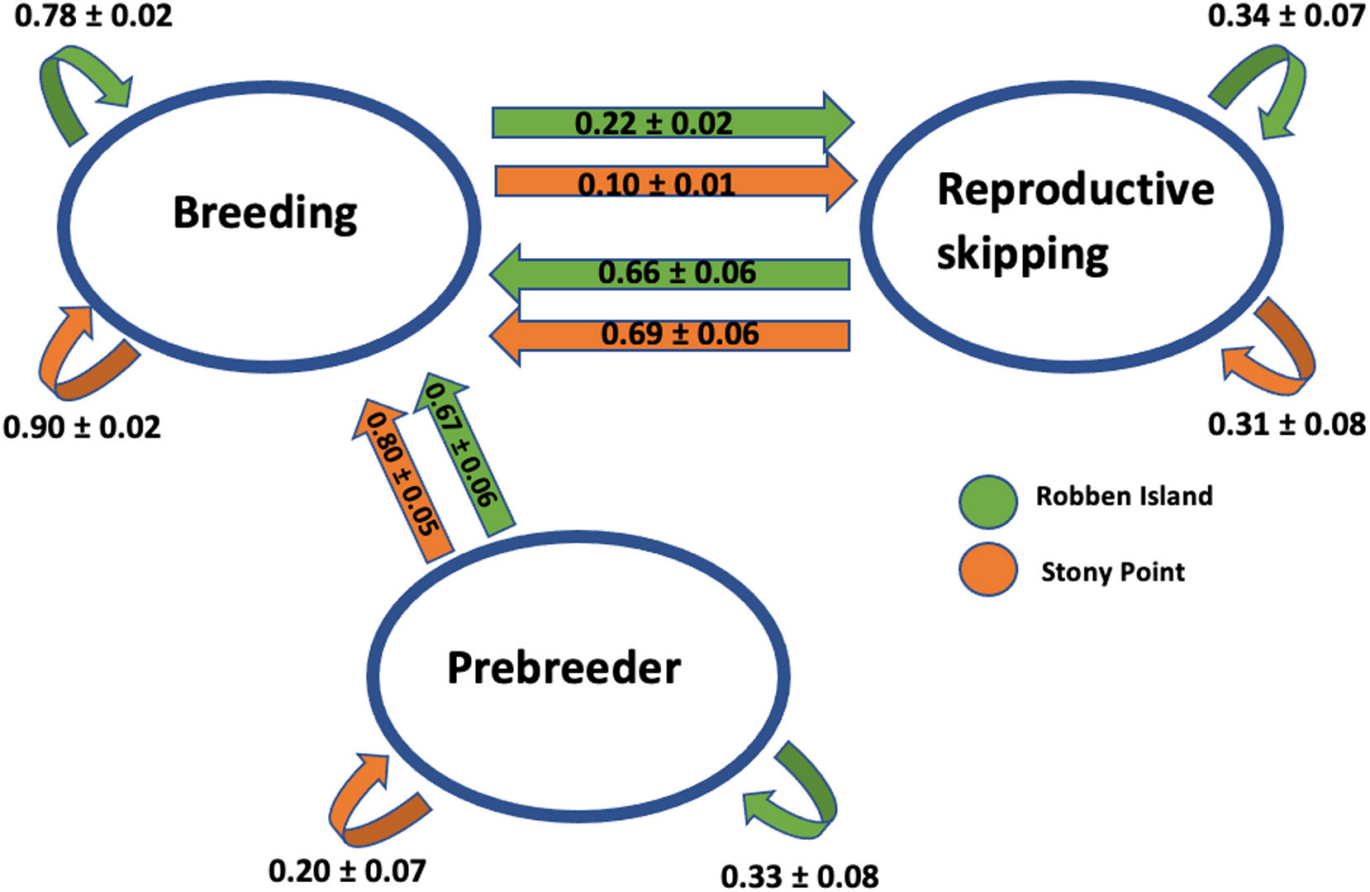
Graphical representation of the probabilities (±*SE*) of transitioning between breeding states in African penguins based on multistate models. Estimates are taken from the constant model (model C30, Table S4).

The recent population trend for African penguins at Stony Point has generally been positive while that at Robben Island has been negative, but the drivers of this difference have not been fully elucidated (Sherley et al., 2020). We identified clear differences in survival and breeding propensity between the two colonies, drivers that likely underpin these divergent population trends. Overall, individuals at Stony Point had higher adult survival and a higher probability of breeding than those at Robben Island, with breeders at Stony Point also more likely to remain in a breeding state and nonbreeding individuals (including prebreeders) more likely to transition into a breeding state (Figure 5). Trends over time indicate this difference is growing, with an increasing presence of reproductive skipping behavior over time at Robben Island (Figure 4a). Notably, the one occasion at Robben Island where breeding individuals were estimated to be more likely to skip reproduction the following year than to remain breeders (Figure 4a) coincided with the lower encounter rates in 2020 (Figure 2). The reduced monitoring effort during the Covid‐19 pandemic may, therefore, have led to more birds being present but not observed, which could have led to an overestimation of reproductive skipping rates in this year.

The presence of variation in breeding propensity over time suggests reproductive skipping here is not purely driven by the individual constraints of lower quality individuals, but implicates external drivers that differ between colonies. In other seabirds, food resources are a prominent driver of reproductive skipping (Gauthier‐Clerc et al., 2001). For example, in king penguins, individuals will abandon breeding attempts when their body mass drops below a certain threshold (Gauthier‐Clerc et al., 2001) and recent research has highlighted higher adult body mass at Stony Point than in nearby Western Cape colonies (Espinaze et al., 2020), which may help us understand the differences in breeding propensity between our two study colonies. Despite this, and previous work linking food availability to lower reproductive output in African penguins (Campbell et al., 2019; Sherley et al., 2013, 2018), this analysis did not find support for food abundance as a driver of reproductive skipping in African penguins. As with the relationship between food availability and survival, fully disentangling this relationship requires future study with colony‐specific measures of prey availability, along with additional years of monitoring to improve the estimates of the transition parameters and better capture the full variation in breeding decisions and how they relate to food availability.

In conclusion, we reveal key insights into African penguin demography, providing the first estimates of reproductive skipping in this species. Penguins are breeding less than theoretically possible, with reduced breeding propensity driven predominantly by individual‐specific constraints. We also present clear inter‐colony differences: individuals at Robben Island are responding more negatively to declining food availability and are characterized by lower survival and a lower breeding propensity than those at Stony Point. These differences highlight a need for a more detailed understanding of the localized drivers of these differences in population dynamics and imply a greater need for conservation action at Robben Island, beginning with actions to improve access to prey.

## AUTHOR CONTRIBUTIONS

**Freddie W. Leith:** Formal analysis (lead), Visualization (lead), Writing – original draft (lead), Writing – review & editing (equal). **Jennifer L. Grigg:** Conceptualization (equal); formal analysis (supporting); investigation (equal); methodology (equal); supervision (supporting); visualization (supporting); writing – review and editing (equal). **Barbara J. Barham:** Data curation (equal); investigation (equal); writing – review and editing (equal). **Peter J. Barham:** Funding acquisition (equal); investigation (equal); writing – review and editing (equal). **Katrin Ludynia:** Data curation (equal); funding acquisition (equal); investigation (equal); writing – review and editing (equal). **Cuan McGeorge:** Investigation (equal); writing – review and editing (equal). **Andile Mdluli:** Investigation (equal); writing – review and editing (equal). **Nola J. Parsons:** Investigation (equal); writing – review and editing (equal). **Lauren J. Waller:** Data curation (equal); investigation (equal); writing – review and editing (equal). **Richard B. Sherley:** Conceptualization (equal); formal analysis (supporting); funding acquisition (equal); investigation (equal); methodology (equal); supervision (lead); visualization (supporting); writing – original draft (supporting); writing – review and editing (equal).

## CONFLICT OF INTEREST

The authors declare no competing interests.

### OPEN RESEARCH BADGES

This article has earned an Open Data badge for making publicly available the digitally‐shareable data necessary to reproduce the reported results. The data is available at This article has earned an Open Data badge for making publicly available the digitally‐shareabledata necessary to reproduce the reported results. The data is available at https://doi.org/10.5061/dryad.0rxwdbs3z.

## Supporting information


Appendix S1

## Data Availability

The data underlying this article are available in the Dryad digital repository: https://doi.org/10.5061/dryad.0rxwdbs3z (Leith et al., 2022).
