# Supplementary material for: Intercolony variation in reproductive skipping in the African penguin
Source: Ecol Evol. 2022 Sep 6;12(9):e9255. doi: 10.1002/ece3.9255 (PMC9448970; doi:10.1002/ece3.9255)
Supplement: Supplementary file 1 — Appendix S1 [file ECE3-12-e9255-s001.docx]

**Appendix S1 – Density Dependence**

One possible explanation for the unexpected negative relationship between sardine biomass and survival of breeders at Stony Point is density dependence. When sardine biomass is high, particularly when it is high west of Cape Agulhas, a higher proportion of adults survive at the relatively large colonies on the west coast of Dassen Island and Robben Island (Sherley et al. 2014; Robinson et al. 2015; Figure 3). Mature adults from Dassen Island (~2,575 pairs in 2013) feed in similar areas on the Agulhas Bank as adults from Stony Point (~2,000 breeding pairs in 2013) during their pre-moult migration (Carpenter-Kling *et al.* 2022). It is likely that birds from Robben Island (~1,350 pairs in 2013), Simon’s Town (between Robben Island and Stony Point, ~600 pairs in 2013), and Dyer Island (south-east of Stony Point, ~1,250 pairs in 2013) also feed in this area. But, post-moult, birds from Dassen Island revert to feeding on the west coast, to the north of Cape Town (Carpenter-Kling *et al.* 2022), likely because they need to access prey resources very rapidly after moulting to rebuild lost body reserves. Therefore, it is possible that per capita pressure on prey resources is higher around Stony Point during the pre-moult period in years when survival rates are better at Dassen and Robben Island, which may result in greater depletion of prey reserves, which then makes it harder for birds at Stony Point to find prey immediately post-moult.

Ideally we would have included regional population size estimates as additional explanatory variables in our CMR modelling to test this idea. However, probably because of the positive relationship between adult survival and sardine biomass (Figure 3), there is a strong positive correlation between our sardine biomass covariate and the annual breeding population size estimates (from Sherley *et al*. 2020) for the west coast colonies (Pearson Pearson’s product-moment correlation: r_p_ = 0.79, t_5_ = 2.90, p = 0.034). Correlations between covariates >0.7 can lead to colinearility issues that can severely distort model estimation, including leading to changes of signs on coefficient estimates (Dorman *et al*. 2013).

However, in support of the idea that density dependence may be a factor in these relationships, the annual survival estimates for breeders (from the best supported time-dependent model B32, Table S4) at Robben Island and Stony Point are strongly negatively correlated (r_p_ = −0.91, t_4_ = −4.40, p = 0.012) and there is a positive correlation between the annual breeding population in the whole of the Western Cape (between 2013 and 2019, from Sherley *et al*. 2020) and our estimates of the probability of birds switching from breeders to nonbreeders at Stony Point (r_p_ = 0.84, t_4_ = 3.12, p = 0.036). In other words, more birds at Stony Point decide to skip breeding as the Western Cape population increases, but this is not the case at Robben Island, where the same correlation is not significant (r_p_ = 0.18, t_5_ = 0.42, p = 0.69). But, neither correlation between our survival rate estimates and either the annual breeding population for the west coast colonies (r_p_ = −0.70, t_4_ = −1.96, p = 0.12) nor the whole Western Cape population (r_p_ = −0.52, t_4_ = −1.22, p = 0.29) was significant. Though both were strong and in the direction expected (negative relationships).

In short, we can neither confirm or rule out an influence of density dependence on reproductive skipping and survival with the data we have available in this study, but would highlight this as a focus for future research.

**Appendix S2 – Additional Figures**

**Table S1:** Number of African penguins *Spheniscus demersus* marked each year with passive integrated transponders (PIT tags) in each colony, organised by breeding state.

| **Robben Island** | | | **Stony Point** | |
| --- | --- | --- | --- | --- |
| **Year** | **Breeder** | **Nonbreeder**  **(prebreeder)** | **Breeder** | **Nonbreeder**  **(prebreeder)** |
| **2013** | 44 | 0 | 42 | 0 |
| **2014** | 25 | 0 | 31 | 2 |
| **2015** | 70 | 10 | 77 | 18 |
| **2016** | 58 | 20 | 114 | 63 |
| **2017** | 67 | 29 | 39 | 15 |
| **2018** | 0 | 0 | 0 | 0 |
| **2019** | 45 | 6 | 30 | 0 |
| **Total** | **317** | **70** | **412** | **100** |

**Table S2:** Number of African penguins *Spheniscus demersus* encountered (or marked) each year, organised by breeding state. ‘Breeder’ refers to individuals confirmed breeding during nest inspections, and ‘Probable Breeder’ refers to individuals encountered in the colony via the ground reader ≥ 6 times over a minimum of 12 days and a maximum of 120 days but not confirmed as breeders during nest inspections.

| **Robben Island** | | |  | | **Stony Point** | | |  |
| --- | --- | --- | --- | --- | --- | --- | --- | --- |
| **Year** | **Breeder** | **Probable**  **Breeder** | **Nonbreeder** | **Breeder** | | **Probable Breeder** | **Nonbreeder** | |
| **2013** | 44 | 0 | 0 | 42 | | 0 | 0 | |
| **2014** | 43 | 1 | 4 | 31 | | 2 | 36 | |
| **2015** | 106 | 3 | 13 | 112 | | 0 | 23 | |
| **2016** | 111 | 11 | 41 | 114 | | 0 | 73 | |
| **2017** | 125 | 34 | 54 | 39 | | 182 | 38 | |
| **2018** | 69 | 47 | 53 | 19 | | 148 | 46 | |
| **2019** | 107 | 8 | 42 | 34 | | 169 | 21 | |
| **2020** | 41 | 8 | 34 | 100 | | 145 | 19 | |
| **Total** | **646** | **112** | **241** | **491** | | **646** | **256** | |

**Table S3.** Detailed goodness-of-fit results for the general (JMV) model calculated using the Rpackage ‘R2ucare’.

| **Test** | **Stat** | **DF** | **P value** | **Testing for** |
| --- | --- | --- | --- | --- |
| **Test 3G.SR** | 16.3 | 18 | 0.574 | Transience^*^ |
| **Test 3G.SM** | 54.6 | 30 | 0.004 | Transience |
| **Test 3G WBWA** | 16.9 | 9 | 0.05 | Memory effects^†^ |
| **Test M.LTECH** | 6.8 | 3 | 0.078 | Trap dependence^‡^ |
| **Test M.ITEC** | 24.3 | 5 | <0.001 | Trap dependence |
| **Overall** | 115.6 | 65 | <0.001 |  |

**Notes: ^*^**For individuals encountered again, does when they were reencountered differ among previously and newly marked individuals? ^†^Is there a difference in the expected state of the next reencounter among individuals previously encountered in the different states (e.g. if site at time *i* effects site at time *i*+1)? ^‡^Is there among-individual heterogeneity in encounter probability, i.e. a difference in the probabilities of being reencountered in the different states at *i*+1 between the animals in the same state at occasion i whether encountered or not encountered at occasion i, conditional on presence at both occasions? SR/ITEC are short-term effects and SM/LTEC are long-term effects. See Sanz-Aguilar et al. (2008) and Choquet et al. (2009).

**Table S4.** Full model results from the multistate mark-recapture models created in RMARK to assess encounter, survival, and transition (breeding propensity) probabilities for African penguins marked with passive integrated transponders and encountered at Robben Island and Stony Point between 2013 and 2020.

**A: Recapture**

| **Model No.** | **Recapture** | **Survival** | **Transition** | **k** | **QAICc** | **∆QAICc** | **QDeviance** |
| --- | --- | --- | --- | --- | --- | --- | --- |
| A10 | Time*C+St | Time*C*St | Time*C*St | 100 | 35744.49 | 0.00 | 33631.59 |
| A21 | Tsm+St | Time*C*St | Time*C*St | 88 | 35750.86 | 6.36 | 33664.33 |
| A12 | Time+C+St | Time*C*St | Time*C*St | 94 | 35751.10 | 6.61 | 33651.42 |
| A20 | Tsm+C+St | Time*C*St | Time*C*St | 89 | 35751.91 | 7.41 | 33663.19 |
| A15 | Tsm*C+st | Time*C*St | Time*C*St | 90 | 35754.10 | 9.60 | 33663.19 |
| A17 | Tsm*St | Time*C*St | Time*C*St | 90 | 35754.39 | 9.90 | 33663.49 |
| A18 | Tsm*St+C | Time*C*St | Time*C*St | 91 | 35755.61 | 11.12 | 33662.52 |
| A13 | Time+St | Time*C*St | Time*C*St | 93 | 35759.99 | 15.49 | 33662.51 |
| A16 | Tsm*C*St | Time*C*St | Time*C*St | 96 | 35766.55 | 22.06 | 33662.48 |
| A9 | Time*St | Time*C*St | Time*C*St | 105 | 35771.51 | 27.02 | 33647.52 |
| A6 | Time*C | Time*C*St | Time*C*St | 98 | 35778.96 | 34.46 | 33670.47 |
| A8 | Time*C*St | Time*C*St | Time*C*St | 126 | 35793.79 | 49.29 | 33622.60 |
| A14 | Tsm*C | Time*C*St | Time*C*St | 88 | 35808.66 | 64.16 | 33722.13 |
| A2 | Tsm | Time*C*St | Time*C*St | 86 | 35814.60 | 70.11 | 33732.44 |
| A19 | Tsm+C | Time*C*St | Time*C*St | 87 | 35815.83 | 71.34 | 33731.49 |
| A11 | Time+C | Time*C*St | Time*C*St | 92 | 35816.05 | 71.55 | 33720.76 |
| A5 | C+St | Time*C*St | Time*C*St | 88 | 35821.74 | 77.25 | 33735.21 |
| A3 | C*St | Time*C*St | Time*C*St | 90 | 35825.25 | 80.76 | 33734.35 |
| A4 | St | Time*C*St | Time*C*St | 87 | 35832.35 | 87.86 | 33748.01 |
| A7 | Time | Time*C*St | Time*C*St | 91 | 35835.42 | 90.93 | 33742.33 |
| A1 | C | Time*C*St | Time*C*St | 86 | 35957.83 | 213.33 | 33875.67 |

**Notes:** *K* = the number of estimated parameters, QAICc = Akaike Information Criterion corrected for over-dispersion and small sample size, and **∆**QAICc = the difference in QAICc between the model in question and the best supported model. Interactions between parameters are denoted by (*) and additive effects by (+). In each model, t refers to time dependence, C to the group effect for colony, St to state (prebreeder, nonbreeder, breeder), and tsm to the grouping of recapture years by ground reader placement. Combined annual Anchovy and Sardine spawner biomass was referred to as ‘Fish’, and separated data by ‘Anchovy’ and ‘Sardine’ respectively. Recapture models were created with survival and transition in their most general form (time, colony and state dependent). Survival was then modelled using the best recapture model and holding transition in its most general form. Transition models were then modelled using the best recapture and survival model.

**Table S4. Cont.**

**B: Survival**

| **Model No.** | **Recapture** | **Survival** | **Transition** | **k** | **QAICc** | **∆QAICc** | **QDeviance** |
| --- | --- | --- | --- | --- | --- | --- | --- |
| B22 | Time*C+St | Sardine*C | Time*C*St | 62 | 35696.46 | 0.00 | 33665.99 |
| B32 | Time*C+St | Time*C | Time*C*St | 72 | 35697.81 | 1.35 | 33645.95 |
| B24 | Time*C+St | Sardine*C+St | Time*C*St | 64 | 35699.00 | 2.55 | 33664.27 |
| B23 | Time*C+St | Sardine*C*St | Time*C*St | 70 | 35700.61 | 4.15 | 33653.05 |
| B34 | Time*C+St | Time | Time*C*St | 65 | 35701.89 | 5.43 | 33665.02 |
| B38 | Time*C+St | Time+C | Time*C*St | 66 | 35703.38 | 6.93 | 33664.38 |
| B33 | Time*C+St | Time*C+St | Time*C*St | 74 | 35704.18 | 7.72 | 33648.02 |
| B13 | Time*C+St | Fish*C | Time*C*St | 62 | 35704.86 | 8.40 | 33674.39 |
| B10 | Time*C+St | C | Time*C*St | 60 | 35704.95 | 8.50 | 33678.74 |
| B25 | Time*C+St | Sardine | Time*C*St | 60 | 35705.42 | 8.96 | 33679.20 |
| B40 | Time*C+St | Time+St | Time*C*St | 67 | 35705.86 | 9.41 | 33664.73 |
| B7 | Time*C+St | Anchovy+C | Time*C*St | 61 | 35705.91 | 9.45 | 33677.56 |
| B1 | Time*C+St | Anchovy*C | Time*C*St | 62 | 35706.10 | 9.64 | 33675.63 |
| B28 | Time*C+St | Sardine+C | Time*C*St | 61 | 35706.18 | 9.73 | 33677.84 |
| B19 | Time*C+St | Fish+C | Time*C*St | 61 | 35706.43 | 9.98 | 33678.09 |
| B12 | Time*C+St | C+St | Time*C*St | 62 | 35707.04 | 10.58 | 33676.57 |
| B39 | Time*C+St | Time+C+St | Time*C*St | 68 | 35707.24 | 10.78 | 33663.96 |
| B8 | Time*C+St | Anchovy+C+St | Time*C*St | 63 | 35707.41 | 10.95 | 33674.81 |
| B15 | Time*C+St | Fish*C+St | Time*C*St | 64 | 35707.51 | 11.05 | 33672.78 |
| B4 | Time*C+St | Anchovy | Time*C*St | 60 | 35708.03 | 11.57 | 33681.81 |
| B30 | Time*C+St | Sardine+St | Time*C*St | 62 | 35708.09 | 11.63 | 33677.62 |
| B20 | Time*C+St | Fish+C+St | Time*C*St | 63 | 35708.13 | 11.67 | 33675.53 |
| B16 | Time*C+St | Fish | Time*C*St | 60 | 35708.19 | 11.73 | 33681.97 |
| B18 | Time*C+St | Fish*St+C | Time*C*St | 65 | 35708.24 | 11.78 | 33671.37 |
| B26 | Time*C+St | Sardine*St | Time*C*St | 64 | 35708.35 | 11.89 | 33673.62 |
| B3 | Time*C+St | Anchovy*C+St | Time*C*St | 64 | 35708.48 | 12.02 | 33673.75 |
| B29 | Time*C+St | Sardine+C+St | Time*C*St | 63 | 35708.49 | 12.03 | 33675.89 |
| B31 | Time*C+St | St | Time*C*St | 61 | 35708.51 | 12.05 | 33680.16 |
| B6 | Time*C+St | Anchovy*St+C | Time*C*St | 65 | 35708.60 | 12.14 | 33671.73 |
| B27 | Time*C+St | Sardine*St+C | Time*C*St | 65 | 35708.61 | 12.15 | 33671.75 |
| B11 | Time*C+St | Colony*St | Time*C*St | 64 | 35708.88 | 12.43 | 33674.15 |
| B17 | Time*C+St | Fish*St | Time*C*St | 64 | 35709.52 | 13.07 | 33674.79 |
| B5 | Time*C+St | Anchovy*St | Time*C*St | 64 | 35710.11 | 13.65 | 33675.38 |
| B9 | Time*C+St | Anchovy+St | Time*C*St | 62 | 35710.24 | 13.78 | 33679.77 |
| B21 | Time*C+St | Fish+St | Time*C*St | 62 | 35710.56 | 14.10 | 33680.09 |
| B14 | Time*C+St | Fish*C*St | Time*C*St | 70 | 35710.81 | 14.35 | 33663.25 |
| B2 | Time*C+St | Anchovy*C*St | Time*C*St | 70 | 35712.63 | 16.17 | 33665.07 |
| B36 | Time*C+St | Time*St | Time*C*St | 79 | 35722.45 | 26.00 | 33655.50 |
| B37 | Time*C+St | Time*St+C | Time*C*St | 80 | 35724.11 | 27.65 | 33654.98 |
| B35 | Time*C+St | Time*C*St | Time*C*St | 100 | 35744.49 | 48.04 | 33631.59 |

**Table S4. Cont.**

**C: Transition**

| **Model No.** | **Recapture** | **Survival** | **Transition** | **k** | **QAICc** | **∆QAICc** | **QDeviance** |
| --- | --- | --- | --- | --- | --- | --- | --- |
| C36 | Time*C+St | Sardine*C | Time*C*St | 62 | 35696.45 | 0.00 | 33665.98 |
| C34 | Time*C+St | Sardine*C | Time*C+St | 36 | 35727.94 | 31.48 | 33752.08 |
| C30 | Time*C+St | Sardine*C | C*St | 26 | 35731.43 | 34.97 | 33776.20 |
| C3 | Time*C+St | Sardine*C | Anchovy*C*St | 32 | 35734.51 | 38.06 | 33766.93 |
| C6 | Time*C+St | Sardine*C | Anchovy*St+C | 27 | 35736.92 | 40.47 | 33779.64 |
| C13 | Time*C+St | Sardine*C | Fish*C*St | 32 | 35737.15 | 40.69 | 33769.56 |
| C31 | Time*C+St | Sardine*C | C+St | 24 | 35737.32 | 40.86 | 33786.19 |
| C22 | Time*C+St | Sardine*C | Sardine*C*St | 32 | 35737.90 | 41.45 | 33770.32 |
| C39 | Time*C+St | Sardine*C | Time+C+St | 30 | 35738.31 | 41.85 | 33774.85 |
| C27 | Time*C+St | Sardine*C | Sardine+C+St | 25 | 35738.76 | 42.30 | 33785.58 |
| C18 | Time*C+St | Sardine*C | Fish+C+St | 25 | 35739.33 | 42.87 | 33786.15 |
| C16 | Time*C+St | Sardine*C | Fish*St+C | 27 | 35739.35 | 42.89 | 33782.06 |
| C8 | Time*C+St | Sardine*C | Anchovy+C+St | 25 | 35739.37 | 42.91 | 33786.19 |
| C14 | Time*C+St | Sardine*C | Fish*C+St | 26 | 35740.20 | 43.74 | 33784.97 |
| C4 | Time*C+St | Sardine*C | Anchovy*C+St | 26 | 35740.26 | 43.80 | 33785.02 |
| C23 | Time*C+St | Sardine*C | Sardine*C+St | 26 | 35740.39 | 43.93 | 33785.16 |
| C37 | Time*C+St | Sardine*C | Time*St+C | 42 | 35741.40 | 44.95 | 33753.07 |
| C25 | Time*C+St | Sardine*C | Sardine*St+C | 27 | 35741.65 | 45.19 | 33784.36 |
| C29 | Time*C+St | Sardine*C | St | 23 | 35744.48 | 48.03 | 33795.40 |
| C40 | Time*C+St | Sardine*C | Time+St | 29 | 35745.31 | 48.86 | 33783.91 |
| C5 | Time*C+St | Sardine*C | Anchovy*St | 26 | 35745.41 | 48.95 | 33790.18 |
| C28 | Time*C+St | Sardine*C | Sardine+St | 24 | 35746.47 | 50.01 | 33795.34 |
| C9 | Time*C+St | Sardine*C | Anchovy+St | 24 | 35746.47 | 50.01 | 33795.34 |
| C19 | Time*C+St | Sardine*C | Fish+St | 24 | 35746.50 | 50.05 | 33795.37 |
| C15 | Time*C+St | Sardine*C | Fish*St | 26 | 35747.28 | 50.83 | 33792.05 |
| C24 | Time*C+St | Sardine*C | Sardine*St | 26 | 35749.57 | 53.11 | 33794.34 |
| C35 | Time*C+St | Sardine*C | Time*St | 41 | 35751.10 | 54.64 | 33764.84 |
| C33 | Time*C+St | Sardine*C | Time*C | 34 | 35798.06 | 101.60 | 33826.34 |
| C2 | Time*C+St | Sardine*C | Anchovy*C | 24 | 35844.31 | 147.85 | 33893.18 |
| C38 | Time*C+St | Sardine*C | Time+C | 28 | 35844.57 | 148.12 | 33885.23 |
| C12 | Time*C+St | Sardine*C | Fish*C | 24 | 35846.48 | 150.02 | 33895.35 |
| C32 | Time*C+St | Sardine*C | Time | 27 | 35853.20 | 156.74 | 33895.91 |
| C10 | Time*C+St | Sardine*C | C | 22 | 35855.38 | 158.92 | 33908.34 |
| C26 | Time*C+St | Sardine*C | Sardine+C | 23 | 35856.14 | 159.68 | 33907.06 |
| C7 | Time*C+St | Sardine*C | Anchovy+C | 23 | 35856.82 | 160.37 | 33907.74 |
| C17 | Time*C+St | Sardine*C | Fish+C | 23 | 35857.27 | 160.82 | 33908.19 |
| C21 | Time*C+St | Sardine*C | Sardine*C | 24 | 35857.50 | 161.05 | 33906.37 |
| C1 | Time*C+St | Sardine*C | Anchovy | 22 | 35867.86 | 171.40 | 33920.82 |
| C11 | Time*C+St | Sardine*C | Fish | 22 | 35868.76 | 172.30 | 33921.72 |
| C20 | Time*C+St | Sardine*C | Sardine | 22 | 35870.23 | 173.78 | 33923.20 |
